# Household transmission of invasive group A *Streptococcus* infections in England: a population-based study, 2009, 2011 to 2013

**DOI:** 10.2807/1560-7917.ES.2017.22.19.30532

**Published:** 2017-05-11

**Authors:** Rachel Mearkle, Maria Saavedra-Campos, Theresa Lamagni, Martine Usdin, Juliana Coelho, Vicki Chalker, Shiranee Sriskandan, Rebecca Cordery, Chas Rawlings, Sooria Balasegaram

**Affiliations:** 1 Field Epidemiology Services, South East and London, National Infection Service, Public Health England, England; 2 Healthcare-Associated Infection and Antimicrobial Resistance Department, National Infection Service, Public Health England, England; 3 National infection Service, England; 4 Respiratory and Vaccine Preventable Bacteria Reference Unit, Public Health England, England; 5 NIHR Health Protection Research Unit in HCAI and AMR, Imperial College London, England; 6 South London Health Protection Team, Public Health England, England

**Keywords:** Invasive streptococcal infections, disease outbreaks, public health policy, *Streptococcus pyogenes*, Group A *Streptococcus*

## Abstract

Invasive group A streptococcal infection has a 15% case fatality rate and a risk of secondary transmission. This retrospective study used two national data sources from England; enhanced surveillance (2009) and a case management system (2011–2013) to identify clusters of severe group A streptococcal disease. Twenty-four household pairs were identified. The median onset interval between cases was 2 days (range 0–28) with simultaneous onset in eight pairs. The attack rate during the 30 days after first exposure to a primary case was 4,520 per 100,000 person-years at risk (95% confidence interval (CI): 2,900–6,730) a 1,940 (95% CI: 1,240–2,880) fold elevation over the background incidence. The theoretical number needed to treat to prevent one secondary case using antibiotic prophylaxis was 271 overall (95% CI: 194–454), 50 for mother-neonate pairs (95% CI: 27–393) and 82 for couples aged 75 years and over (95% CI: 46–417). While a dramatically increased risk of infection was noted in all household contacts, increased risk was greatest for mother-neonate pairs and couples aged 75 and over, suggesting targeted prophylaxis could be considered. Offering prophylaxis is challenging due to the short time interval between cases emphasising the importance of immediate notification and assessment of contacts.

## Introduction

Group A *Streptococcus* (GAS) causes a range of illnesses, from the relatively mild pharyngitis to severe, life-threatening disease [1]. Invasive GAS (iGAS) infection has a case fatality rate exceeding 15% [2] rising to 25% in resource-limited countries [1]. In the United Kingdom (UK), iGAS has an estimated incidence rate of 3.33 per 100,000 population per year [3].

Only four population-based studies have been published quantifying the risk of secondary household transmission, describing a total of 13 household clusters across four countries (from Australia, Canada, UK and the United States (US)) and identifying a considerable increased risk of transmission in household contacts [2,4-6]. However, quantification of the absolute risk and the most appropriate public health response to a single case of iGAS infection is problematic. International guidance on antibiotic prophylaxis for contacts after a single case of iGAS infection varies across countries; the UK [7] and Ireland [8] recommend prophylaxis of household contacts only in specific circumstances, namely for post-partum mothers and neonates where the other develops iGAS infection. Other contacts are systematically followed up to inform them of their increased risk and advised to seek medical care if they have symptoms of GAS infection or develop them within 30 days from exposure [7,8]. In France [9] and the US [10] chemoprophylaxis is recommended for those identified as having an increased risk of infection. The Public Health Agency of Canada recommends selective prophylaxis for household contacts when a case is fatal or presents with severe symptoms including streptococcal toxic shock syndrome, soft-tissue necrosis, meningitis or pneumonia [11].

In order to inform the revision of the current UK guidelines, national surveillance data from 2009, 2011–2013 in England were used to estimate the risk of secondary transmission in household contacts of iGAS cases.

## Methods

### Data sources

We examined two different national datasets for England to identify incidents of secondary household transmission. From 1 January 2009 to 31 December 2009 online enhanced surveillance questionnaires were completed jointly by hospital microbiologists and Health Protection Teams (HPTs). HPTs undertake local disease surveillance and investigate and manage local health protection incidents. Enhanced surveillance was initiated in 2009 due to an increase of iGAS infection notifications in England [12].

From 1 January 2011 to 31 December 2013, we used data from the newly introduced HPTs web-based case and incident management system HPZone (inFact Shipley Ltd 2012). Data from 2010 were excluded as the HPZone system was implemented that year and it was not possible to confirm that all data were captured and although iGAS infection became a notifiable disease in England from 2010 [13], data were likely to be incomplete. No ethical approval was required as only routinely collected data were analysed.

The two data sources collected demographic and clinical details of cases; however not all data required for this study were available in both datasets. Information on risk factors for transmission was only systematically collected on the enhanced surveillance questionnaires and detailed case management data were only available from HPZone. Data from the north-east of England were not made available for 2011 to 2013 for this study; the results have been adjusted for this.

### Case definitions

A confirmed case was defined as an individual with an infection associated with the isolation of GAS from a normally sterile site or a non-sterile site in a patient with a severe clinical presentation (streptococcal toxic shock, necrotising fasciitis, pneumonia, puerperal sepsis, septic arthritis, meningitis, peritonitis, osteomyelitis, or myositis).

A probable case was defined as an individual where GAS was not isolated but the case was epidemiologically linked to a confirmed case and had a severe clinical presentation consistent with iGAS disease (as described above).

A household cluster comprised of two or more cases (probable or confirmed) with onset of illness within 30 days of each other and where the cases were known to have had direct person-to-person contact with each other in a household, in the 7 days before onset of illness of the primary case.

A primary case was defined as the first case admitted to hospital as part of a household cluster.

Secondary case(s) included the subsequent case(s) admitted to hospital as part of a household cluster.

Co-primary cases were defined as two or more cases from the same household who were admitted to hospital within 24 hours of each other.

Cases whose infection was acquired in any non-household setting, including hospitals, care homes and hostels, were excluded from the investigation.

### Cluster identification

Household clusters were retrospectively identified in the two datasets using postcode matching to identify co-located cases occurring within 30 days of each other. We also reviewed and matched postcodes with isolate referral data and data from the GAS cluster database held by the Respiratory and Vaccine Preventable Bacterial Reference Unit, Public Health England, to identify clusters previously unidentified. If clusters were identified through reference laboratory data, the corresponding case records (enhanced surveillance questionnaire or HPZone web-based surveillance records) were obtained through local Field Epidemiology Services. Case records were checked to confirm if cases were resident in the same household in the previous 30 days. If clarification of any case details was required, the responsible HPT was contacted to obtain detailed case management notes.

### Microbiological characterisation

In England, *emm* gene sequencing [14] is used in the analysis of GAS clusters and outbreaks to support epidemiological data linking patients. The strains in this study were characterised by sequence analysis of the *emm* gene [15] and compared against a comprehensive database of *emm* types observed from iGAS strains from England submitted during the same time period.

### Statistical analysis

We used STATA 13 (StataCorp LP 2013) to clean and analyse data. We described clinical characteristics and compared sporadic and cluster cases using chi-squared and Fisher’s exact tests. All population estimates were from the Office for National Statistics (ONS) including household size estimates [16]. Subgroup analysis of mother neonate pairs and elderly couples (where both individuals were aged 75 years and over) was performed, including univariate analysis. Data on the proportion of all cases with recent childbirth was estimated from the enhanced surveillance data for 2009 and extrapolated to the entire study period to provide an estimated denominator. Data on the number of individuals aged 75 years and over living in a household with another individual aged 75-years-old or older was provided by the ONS for 2011–2013 and extrapolated to the entire study period to provide an estimated denominator. Subgroup analysis included calculation of numbers needed to treat (NNT) using the substitution method [17]; this gives an estimate of the numbers who would need to be offered prophylaxis to prevent one secondary case, assuming 100% adherence and effectiveness of prophylaxis.

## Results

### Description of clusters

We identified 24 household clusters, all of which were pairs of cases (Table 1).

**Table 1:** Description of confirmed and probable cases of invasive group A *Streptococcus* infection, and identified household clusters, England, 2009, 2011–2013 (n = 24 clusters)

Category	Findings for 24 clusters
Classification of cases	• 48 cases: o 35 confirmed iGAS cases o 10 confirmed severe GAS cases o 3 probable severe GAS / iGAS cases • 12 iGAS pairs; GAS isolated from a sterile site in both cases • 12 severe GAS pairs: at least one case where GAS was isolated from a non-sterile site (9 pairs) or a probable case (3 pairs)
Clinical presentation	• 16 co-primary cases: 3 presented with upper and/or lower respiratory disease^a^, 3 with skin and soft tissue infection (SSTI)^b^, 10 with sepsis from an unknown or other focus • 16 primary cases: 5 presented with respiratory disease^a^, 8 with skin and soft tissue infection^b^, 1 with septic arthritis and 2 with an unknown or other focus • 16 secondary cases: 4 presented with respiratory disease^a^, 6 with skin and soft tissue infection^b^, 1 with meningitis, 1 with septic arthritis and 4 with an unknown or other focus
Relationship of cases	• 8 partner/spouse pairs (6 with both aged 75 years and over) • 5 mother and neonates (0–10 days old) • 4 child siblings (1–9 years old) • 3 parent and child (4–13 years old) • 2 young adult injecting drug users • 2 parent and adult child pairs
Outcome of infection	• For 8 cases GAS was the cause or contributing cause of death (17% fatality rate) o 2 of 16 cases in unrelated co-primary cases (13% case fatality) o 3 of 16 primary case (19% case fatality) o 3 of 16 secondary cases (19% case fatality) o 2 of 24 pairs had fatal primary and secondary cases • 8 additional cases were admitted to intensive care units or high dependency units, but were not fatal
Chronic or predisposing risk factor	• 26 cases had risk factors predisposing to iGAS infection including age (75 years and over or under 1 year), injecting drug use, hepatitis C, cancer, diabetes, cardiac disease, and immune deficiency
Acute risk factors	• 17 cases had an acute risk factor; including recent respiratory tract infection, skin infection, wound, or insect bite
*emm* type	• Microbiologically-confirmed GAS: 44 cases • *emm* type available: 35 cases • *emm* type identical in both cases in each pair: 12 pairs • *emm* type only available from one case in the pair: 11 pairs (if the other case was confirmed no *emm* type was available) • *emm* type not available on either case in a pair: 1 pair • More details of the results are presented in Figure 1

GAS: group A *Streptococcus*; iGAS: invasive group A *Streptococcus*; SSTI: skin and soft tissue infection.

^a^ Respiratory disease describes an infection in a patient with a record of pneumonia, empyema, pharyngitis, scarlet fever or influenza-like symptoms in case records as a presentation or acute risk factor.

^b^ SSTI describes an infection in a patient with a record of myositis, necrotising fasciitis, abscess, wound infection, cellulitis, erysipelas, or skin lesion in case records as a presentation or acute risk factor.

Source: 2009 Enhanced surveillance questionnaire, 2011–2013 HPZone web-based surveillance records.

The clusters were geographically spread across all regions in England. In 2009, an additional 15 case records reported a link to another GAS case. We sought further verification from all sources but no linked confirmed or probable cases could be identified and these were therefore excluded from the study.

The most common relationship between cases within a cluster was partners/spouses which accounted for eight of the 24 pairs identified. Half (n = 12) of the pairs involved an individual under 19 years, including five mother neonate pairs. Two pairs were individuals injecting drugs who were not related. The most commonly reported first clinical presentations were skin and soft tissue disease (17 of 48) and respiratory disease (12 of 48) with a further 16 cases having reported sepsis of other or unknown focus. A higher proportion of primary cases had a respiratory presentation (5 of 16) compared with secondary cases and co-primary cases (7 of 32), however this was not statistically significant (chi-squared test p = 0.5). Seventeen cases had an acute risk factor but none had a recent history of chickenpox.

**Figure 1:**
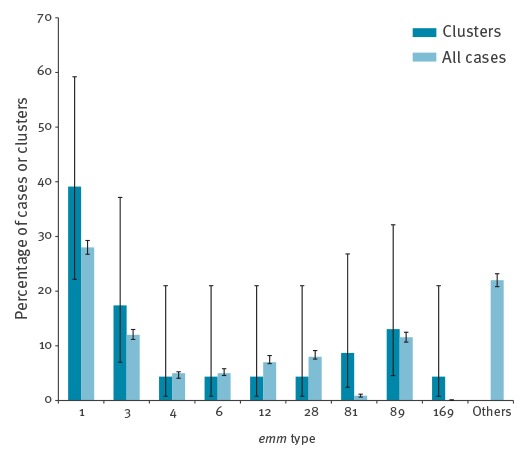
Distribution of *emm* types of all iGAS infection cases and those identified from clusters, England, 2009, 2011–2013 (clusters n = 23, all cases n = 4,889)

Overall most clusters were associated with *emm* 1 infection (Figure 1).

iGAS: invasive group A *Streptococcus*.

Sources: 2009 Enhanced surveillance questionnaire, 2011–2013 HPZone web-based surveillance records and database of *emm* type observed from iGAS strains from England.

This was the most common *emm* type isolated in all iGAS cases during the study period and historically in England. During 2009, *emm* type 3 was the most common in cluster cases and for all cases. Two clusters with less common sequence types were also noted; *emm* type 81 and 169, accounting for two and one of the clusters respectively.

### Time between cases

**Figure 2:**
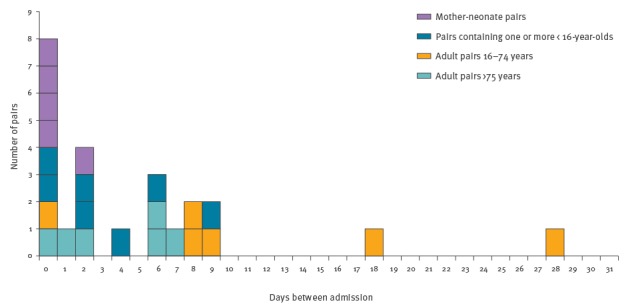
Time between hospital admission of primary and secondary iGAS infection cases, England, 2009, 2011–2013 (n = 24 pairs)

The median number of days between hospital admission of the primary case and diagnosis of the secondary case was two days (range: 0–28 days). The period of risk was concentrated in the first ten days (Figure 2) with eight cases presenting on the same day as the primary case (co-primary cases).

iGAS: invasive group A *Streptococcus*.

**Figure 3:**
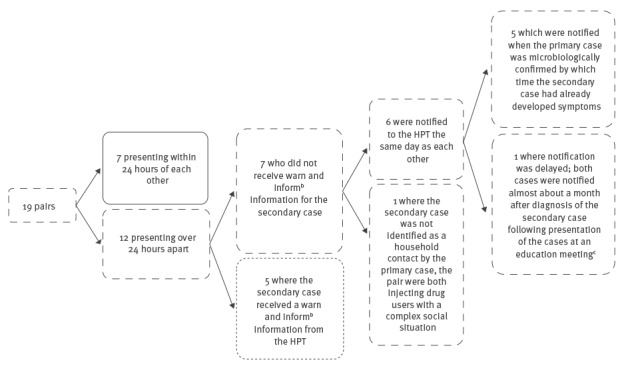
Public health management of iGAS infection clusters, England, 2011–2013^a^

GAS: group A *Streptococcus*; iGAS: invasive group A *Streptococcus;* HPT: Health Protection Team.

Seven of the 19 pairs from 2011 to 13 were co-primaries (Figure 3) as they presented within 24 hours of each other. Only five of 12 pairs that were not co-primaries were contact traced as per national guidelines. Of the remaining seven, one case failed to identify the secondary case as a contact and of the others, five were promptly notified once microbiological diagnosis in the primary case was confirmed and one was delayed due a failure to notify the primary case promptly (Figure 3).

Solid black outline indicates public health actions could not be taken, dashed outline are cases where public health actions could potentially have been taken, and dotted outline where public health actions were taken.

^a^ No information was available on the 2009 cases.

^b^ Warn and inform information indicates that contacts were systematically followed up to inform them of their increased risk, and advised to seek medical care if they had symptoms of GAS infection or develop them within 30 days.

^c^ Education meeting to present and review interesting cases for education purposes.

Source: HPZone web-based surveillance records.

### Risk factors for transmission

Analysis of risk factors was completed on 1,138 cases from enhanced surveillance in 2009, among which there were five clusters. Secondary cases in clusters were younger than sporadic cases (median age 5 years (range: 0–85) vs 50 years (range: 0–99), p = 0.2), accounted for by two of the five clusters being mother-neonate pairs (Table 2).

**Table 2:** Differences between sporadic and secondary cases of iGAS infection, England, 2009 (n = 1,138)

Description	Data completeness (%)	Secondary cases and neonates^a^		All sporadic cases (including primary cases)			Sporadic cases in multi-occupancy households		
		Number	Proportion or range	Number	Proportion or range	p value	Number	Proportion or range	p value
**Number**	NA	5	NA	1,133	NA	NA	333	NA	NA
**F:M**	98	1:0.7	NA	1:1.1	NA	0.7^b^	1:1.1	NA	0.7 ^b^
**Median age**	99	5	0–85	50	0–99	0.2^c^	39	0–93	0.5^c^
**Median household size**	43	5	4–5	2	1–13	0.03^c^	3	2–13	0.07 ^c^
**Number mothers or neonates**	86	2	40%	58	5%	0.03^b^	24	7%	0.05 ^b^
**Number 75 years and over**	99	1	20%	253	22%	1.0^b^	41	12%	0.5 ^b^
**Number with preceding URTI^d^**	60	1	20%	274	24%	1.0^b^	102	31%	1.0 ^b^
**Number with any chronic comorbidity^e^**	86	2	40%	406	36%	1.0 ^b^	104	31%	0.7 ^b^
**Number with acute risk factor ^f^**	86	2	40%	461	41%	1.0 ^b^	140	42%	1.0 ^b^

iGAS: invasive group A *Streptococcus*; NA: not applicable; URTI: upper respiratory tract infection

^a^ Where a mother and neonate co-presented the neonate has been analysed as a secondary case.

^b^ Fishers exact test.

^c^ Kruskall-Wallis chi-squared.

^d^ Recorded pharyngitis/tonsillitis, scarlet fever or influenza-like illness.

^e^ Recorded diabetes, malignancy, chronic respiratory condition, steroid use, dementia, heart disease, homelessness, alcoholism, renal disease, injecting drug use, immunosuppression.

^f^ Recorded skin wound or lesion including surgery, trauma, infection, chickenpox, eczema, pressure sore and impetigo.

Source: Enhanced surveillance questionnaire, Public Health England (at the time Health Protection Agency), London, United Kingdom.

Being a mother or neonate in the post-natal period was a significant risk factor for secondary transmission for both mother and neonates (risk ratio (RR): 11.9; 95% confidence interval (CI): 2.0–70.3). This was based on the 60 recorded cases of infection in post-partum mothers or neonates, two cases were identified to have transmitted in the household. Households with clusters had significantly higher numbers of occupants than those of sporadic cases with a median household size of 5 (range: 4–5) compared with 2 (range: 0–13) in all sporadic cases (p = 0.03) and 3 (range: 2–13; p = 0.07) in multi-occupancy households with sporadic cases, but due to poor data completeness (43% response rate) of household size, results should be interpreted with caution.

A total of 520 (46%) cases were found to have critical markers of severity (Table 3); this included three cluster cases from the five clusters identified in this period. No significant association was identified between severity of the primary case and the likelihood of transmission in the household (RR: 1.8; 95% CI: 0.3–10.6; p = 0.7), however we could not adjust for age due to co-linearity. We also assessed risk based on clinical presentation of the primary case. Three primary cluster cases had respiratory disease (3 of 5, 60%) compared with 34% of all non-cluster cases (380 of 1,133); although, potentially important, this difference did not reach statistical significance (RR: 3.0; 95% CI: 0.5–17.6; p = 0.3).

**Table 3:** Differences between primary cases within a household cluster and sporadic cases of iGAS infection, England, 2009 (n = 1,138)

Description	Primary cases within cluster^a^	All cases which did not produce subsequent case (including secondary cases of clusters)	p value	Risk ratio (95% CI)
**Number**	5	1,133	NA	NA
**Median age**	32	50	p = 0.5^b^	NA
**Cases with critical markers^c^**	3 (60%)	517 (46%)	p = 0.7^d^	1.8 (0.3–10.6)
**Number of deaths where GAS was the main or contributory cause (%)**	2 (40%)	195 (17%)	p = 0.2^d^	3.2 (0.5–18.9)
**Respiratory disease^e^**	3 (60%)	380 (34%)	p = 0.3^d^	3.0 (0.5 – 17.6)

CI: confidence interval; GAS: group A *Streptococcus*, NA: not applicable.

^a^ Where a mother and neonate co-presented, the mother has been analysed as the primary case.

^b^ Kruskall-Wallis chi squared.

^C^ Critical markers are cases that meet the Canadian definition of severity including streptococcal toxic shock syndrome, soft-tissue necrosis (including necrotising fasciitis and myositis), meningitis, pneumonia, Intensive Treatment Unit admission or cause or contributing cause of death at 7 days [11].

^d^ Univariate analysis.

^e^ Respiratory disease if cases reported influenza-like symptoms, pharyngitis, or scarlet fever as a preceding illness, or reported a focus of infection of pharyngitis, pneumonia or empyema.

Source: Enhanced surveillance questionnaire, Public Health England (at the time Health Protection Agency), London, United Kingdom.

### Secondary attack rate

The attack rate during the 30 days post exposure was calculated as 4,520 per 100,000 person-years at risk (95% CI: 2,900–6,730) (Table 4).

**Table 4:** Estimate of attack rate, risk ratio and NNT among household contacts of iGAS infection in England, 2009, 2011–13 (n = 48)

Population	Cases in contacts	Attack rate /100,000	95% CI	Risk ratio	95% CI	Background incidence rate/100,000^a^	NNT	95% CI	Cases excluding co-primary	NNT excluding co-primary	95% C
**Total** **(all ages, all years)**	24	4,520	2,900–6,730	1,940	1,240–2,880	2.34	271	194–454	16	407	273–807
**Mothers and neonates during neonatal period**	5	24,310	7,890–56,740	4,990	1,580–12,330	4.87^b^	50	27–393	1	257	NA
**Couples aged 75 years and over**	6	15,000	5,510–32,650	1,650	600–3,600	9.09^c^	82	46–417	5	98	53–826
**All (excluding mothers and neonates and couples aged 75 years and over)**	13	2,900	1,540–4,960	1,390	740–2,380	2.09	423	274–938	10	552	340–1,478
**Cases who presented over 24 hours apart**	16	3,020	1,720–4,900	1,290	740–2,100	2.34	407	273–807	NA	NA	NA
**iGAS in sterile site clusters only**	12	2,260	1,170–3,950	970	500–1,690	2.34	545	347–1,277	6	1,104	607–6,154

CI: confidence interval; NA: not applicable; NNT: number needed to treat; ONS: Office for National Statistics.

^a^ We estimated the background incidence using the proportion of cases that were residents in the community and acquired iGAS in the community which was 90.95% of all iGAS cases in the study period (unpublished data, M. Saavedra-Campos, March, 2015). The estimated proportion excluded care home residents and hospital acquired cases.

^b^ Calculated using ONS maternity data, this represents the estimated risk for the mother and neonate during the month after birth.

^c^ Background incidence for all 75 year-olds and over.

Figures rounded to three significant digits.

Source: 2009 Enhanced surveillance questionnaire, 2011–2013 HPZone web-based surveillance records, Public Health England, London, United Kingdom.

This equates to an estimated 1,940 (95% CI: 1,240–2,880) -fold elevation over background incidence during this 30-day period. The theoretical number NNT (assuming antibiotic prophylaxis would be 100% effective and could be given in time to prevent all secondary cases) was 271 (194–454). Excluding eight co-primary pairs, we analysed the 16 pairs that had potential time for intervention giving an NNT of 407 (95% CI: 273–807) assuming that contacts could be identified and notified promptly.

Subgroup analysis was undertaken on the two most common types of relationships identified in the pairs: mother-neonates and couples aged 75 and over (including co-primary pairs). We estimated the attack rate for mother-neonate pairs as 24,310 per 100,000 person-years at risk (95% CI: 7,890–56,740), with a theoretical NNT of 50 (95% CI: 27–393). The risk to cohabiting couples aged 75 and over was also elevated with a theoretical NNT of 82 (95% CI: 46–417). Excluding these two groups, the theoretical NNT for the remaining population was 423 (95% CI: 274–938). For three of the five elderly couple pairs identified in 2011–2013, there was sufficient time between notification of cases for public health action to be taken and a contact tracing to be undertaken. Two of the secondary cases in these three pairs were fatal. We estimated the risk of transmission when a 0–18 year-old was the primary case with a NNT of 786 (95% CIs not calculable because they cross 0, n = 3, excluding co-primary clusters). It was not possible to estimate the NNT for risk of transmission to children, due to poor completion of data on household size and composition.

## Discussion

Our study found that the risk of transmission of iGAS infection is substantially elevated in households after a single case, particularly for mothers and neonates during the neonatal period, and for couples aged 75 and over. The attack rate in household contacts was over 4,000 per 100,000 person-years at risk rising to over 25,000 and 15,000 for mothers and neonates and couples aged 75 years and over, respectively. Four previous population studies have estimated the risk of transmission in households [2,4-6]. These gave a pooled estimated attack rate of 2,681 per 100,000 person-years at risk (95% CI: 1,428–4,585) during the 30 days after exposure [5]. This is elevated from the background incidence rate in the four countries, estimated to be between 2 and 4 per 100,000 persons per year in developed countries [18]. This study has a higher estimated attack rate than most of the previous studies (at 4,524), possibly due to the inclusion of individuals with severe GAS infection within the case definition. If we had used a strict iGAS definition (cases microbiologically confirmed from a sterile site) and included only 12 clusters in the analysis, the attack rate was 2,262 per 100,000 person-years at risk (95% CI: 1,169–3,951), consistent with other studies. However we included all severe GAS cases as the case fatality rate (17% observed in this study) is as high as for iGAS infection, thus justifying similar intervention. International recommendations on management of contacts of iGAS infection vary [7-11]. Our findings did not find sufficient evidence to support the use of selective prophylaxis based on severity of primary cases as recommended in Canadian guidelines [11]. Although published case reports describe transmission from respiratory infections in the community and in hospital [19-23], our findings provide some weak evidence to support this but did not reach statistical significance.

Our theoretical NNT for household contacts was 271 (95% CI: 194–454); this is similar to the estimated NNT for meningococcal disease which is 284 (95% CI: 156–1,515), this accounts for the effectiveness of prophylaxis with a risk ratio of 0.14 in those given antibiotics [24]. Estimates for meningococcal disease come from four observational trials, and European Centre for Disease Prevention and Control (ECDC) grades this as ‘moderate quality’ evidence to recommend prophylaxis to household contacts [24]. Meningococcal disease shares a similar risk of transmission in households [25] and both infections carry a notable case fatality rate but epidemiological patterns differ [26]. However, unlike meningococcal disease, our NNT for iGAS is theoretical as it does not take into account the efficacy of chemoprophylaxis, compliance or the effectiveness of implementation of a prophylaxis regime.

This study includes the first published subgroup analysis of household clusters. This has confirmed that mothers and neonates were at particularly high risk of transmission with an NNT of 50, supporting the existing UK guidance recommendations to offer prophylaxis if the case is a mother or a neonate during the neonatal period [7]. Although previous guidance (which advises prophylaxis for mothers and neonates) has been in place since 2004, our study highlights that mothers and neonates still account for one in five household clusters. Case series data suggest that guidance for prophylaxis is not always followed [27]. Insufficient information was available on the five mother neonatal pairs in our study to determine if they received prophylaxis. It is however encouraging that none of the mother or neonatal cases had severe clinical presentations recorded and no deaths were associated within these pairs, perhaps suggesting that current guidance is having some positive impact.

We identified that couples aged 75 years and over were at increased risk, with an NNT of 82. Background incidence and case fatality rate associated with iGAS increase with age [28,29]. There was sufficient time for public health action for the majority of elderly pair clusters, supporting a recommendation that they should be considered for chemoprophylaxis. We do not have data to estimate if the risk is elevated in all aged 75 years and over.

Of the 12 pairs where there was time for public health interventions, only five were contact traced before the secondary case developed symptoms. There needs to be heightened awareness among clinicians of the necessity to promptly notify local public health teams to ensure timely management of patient contacts, similar to that achieved for meningococcal infection [30]. Given the time delay inherent in microbiological culture, use of molecular testing for rapid diagnosis of suspected iGAS infection may reduce delay in notification. As in the case of meningococcal disease, cases should be notified on clinical suspicion so that close contacts can be identified early and advised to be vigilant for symptoms of GAS infection. Our study also highlights the importance of detailed contact tracing particularly around cases with complex social circumstances such as drug use. Recent outbreaks further stress the importance of effective contact tracing in this community [31].

The subgroup analysis was based on only five clusters in mothers and neonates and six clusters in couples aged 75 years and over. Although we are unlikely to have missed any postcode matched cases with triangulation of routine electronic laboratory reports, our data may still not be complete due to cases of severe GAS that have, in error, not been notified to the HPTs, cases who have been in close household contact but have different (or missing) registered addresses. We have included in this study mother and neonate pairs who did not have a case record entered for the neonate, but others may have been missed. Thus, the number of clusters found in this study should be considered a minimum. Reliance on routinely-collected data, with varying levels of completeness, may have led to underestimation of acute and chronic risk factors in our clusters. We have not been able to calculate the risk of transmission from non-invasive disease because our surveillance systems do not capture all GAS disease manifestations. We have used the average household size [16] to calculate the population at risk which cannot distinguish between higher and lower risk households. We have been able to calculate attack rates for mother neonate pairs and elderly couples however it has not been possible to calculate the risk for children, who also account for a large proportion of our cluster cases. Demographic data on household contacts of sporadic cases is not consistently collected and this is an area which could be addressed in future research. This study includes data from 2009 which was unusual due to the increase in *emm* 3 iGAS and data presented here show that *emm* 3 was the most common in cluster cases and for all cases reflecting the upsurge of *emm* 3 noted in England in 2009 [32]. However, this period accounts for 25% (n=1 year) of the study period and 21% (n=5 household clusters) of the clusters identified, and therefore we feel this is unlikely to have significantly influenced the findings of our study. The majority of clusters detailed in this study were associated with *emm* 1 gene strains, reflecting the proportion of invasive disease cases seen nationally. Clusters with two less commonly noted types were also identified. It is possible that specific *emm* gene type lineages have increased capacity for transmission, invasion, cell association and progression to invasive infection conferring increased ability to cause localised clusters of infection such as those described in this study.

The data demonstrate that there is an increased risk of secondary transmission, commensurate to that of meningococcal disease. There is a short time interval between notifications of cases making prevention of secondary cases challenging. Nevertheless secondary cases had a 19% case fatality rate, and given the particularly high risk of transmission for mother and neonate pairs and couples aged 75 years and over, careful consideration should be given to offering routine prophylaxis to these groups. There is a lack of evidence for the efficacy of prophylaxis and more evidence is needed. Efforts should be made to lower the threshold for action (including warn and inform education) and improve notification, despite potential challenges for timely actions.
